# Expectations about the “Natural Order of Things” and Conspiracy Beliefs about COVID-19

**DOI:** 10.3390/ijerph19159499

**Published:** 2022-08-02

**Authors:** Mauro Giacomantonio, Valerio Pellegrini, Valeria De Cristofaro, Maurizio Brasini, Francesco Mancini

**Affiliations:** 1 Department of Social and Developmental Psychology, Sapienza University, 00185 Rome, Italy; valeria.decristofaro@uniroma1.it; 2 Associazione Scuola di Psicoterapia Cognitiva (APC-SPC), 00185 Rome, Italy; brasini@apc.it (M.B.); f.mancini@unimarconi.it (F.M.); 3 Department of Human Sciences, Marconi University, 00193 Rome, Italy

**Keywords:** COVID-19, conspiracy beliefs, expectations

## Abstract

The COVID-19 pandemic represents an event that unsettled the social and economic life of many people. When individuals are faced with shocking events, they may need to find plausible explanations for such events to restore control and make sense of reality. The adoption of conspiracy beliefs may represent a functional strategy for this purpose. The present study investigated whether the endorsement of conspiracy beliefs may be associated with the degree to which an upsetting event (i.e., the COVID-19 pandemic) is perceived as incoherent with individuals’ general set of expectations about the world functioning (i.e., the natural order of things). Analyzing data from a community sample of 565 Italian participants, a path analysis model highlighted a mediation pattern where the natural order of things was negatively related to the adoption of conspiracy beliefs and, thus, was indirectly and positively related to support for the norms aimed at containing the spread of COVID-19, feelings of guilt about neglecting such norms, and intentions to be compliant with COVID-19 vaccination. Moreover, the natural order of things was indirectly and negatively related to attitudes focused on economic issues rather than public health and to negative attitudes towards COVID-19 vaccines through reduced beliefs in conspiracies.

## 1. Introduction

The COVID-19 pandemic constituted a catastrophic global event, which dramatically affected the physical and mental health of millions of individuals all over the world. In addition to the direct impact on health, the pandemic deeply and negatively affected our societies by upsetting, among other things, trade and industry, education, interpersonal relationships, and social life [1,2,3]. Threats concerning health and material welfare were thus accompanied by the crumbling of shared worldviews concerning the way things work. As a result, feelings of fear and uncertainty were raised [4].

Growing empirical evidence suggests that, in order to deal with these aversive feelings, conspiratorial explanations concerning COVID-19 and its treatments (with a particular emphasis on vaccines) spread and were widely endorsed in the population [5,6,7,8].

In the present work, we examined how conspiracy thinking could be endorsed as a compensatory control strategy to address fear and uncertainty linked to the perception that something outside of the “natural order of things” is happening. As we discuss later, events that are perceived as incompatible with the natural order of things do not necessarily have dramatic, personal, and direct consequences. However, they have the potential to destabilize one’s view of the world, thus undermining one’s epistemic expectations and sense of security. Conspiracy beliefs might help to address this sense of violation of the natural order by attributing the responsibility of the event to a certain group of individuals.

### 1.1. COVID-19 Conspiracy Beliefs and Their Consequences

COVID-19 was created in a laboratory for biological warfare purposes. The cure for COVID-19 is already in existence, but big pharma is interested in keeping it secret. COVID-19 vaccines are harmful and useless, and they are part of a strategy to control people’s minds.

These are examples of conspiracy beliefs that emerged in different phases of the pandemic. Although some of them can be regarded as particularly unrealistic, they can have a meaningful negative downside at individual and social levels [6]. The endorsement of conspiracy beliefs has been associated with increased apathy, hostility, prejudice, and reduced pro-sociality [9,10,11]. From a collective point of view, conspiracy thinking can motivate distrust and a strong sense of suspicion towards governments and policymakers, leading people to refuse recommendations and regulations even when they are based on scientific evidence [12]. For instance, it has been documented that advocating conspiratorial accounts of HIV increased risky behavior and decreased compliance with medical treatment [13].

Unsurprisingly, similar effects have also been documented by burgeoning research about conspiracy theories concerning COVID-19 and the related vaccines. Pummerer et al. (2021) [8] found that conspiracy beliefs are associated with reduced institutional trust and support for governmental regulations (see also Pellegrini et al., 2021 [7], for similar findings). Relatedly, intentions to vaccinate are negatively associated with believing in conspiracy theories [14,15,16].

These findings could be explained by considering that, for those who believe in conspiracies, ignoring rules advanced by a malicious system is a worthy behavior. As a rule of thumb, embracing the opposite direction than the one indicated by the system could be wise. This remains true even when severe risks for one’s health stem from the refusal of vaccines or from the choice of using treatments that are not scientifically and empirically justifiable, such as the use of chloroquine against COVID-19 [17].

Recent theorizing emphasized the role of unmet needs in explaining the circumstances under which believing in conspiracy is more likely [6,18]. For example, epistemic needs related to avoiding uncertainty and to perceiving a sense of predictability of reality, as well as existential needs associated with dealing with fears, death, and lack of power can motivate people to refer to conspiracy beliefs [7,19]. This happens because, as we describe in detail later, conspiratorial accounts help to restore a clear-cut and a simplified reading of reality, in which control and agency are reinstated.

In the present research, we want to complement previous work by advancing that perceiving the pandemic as an event that is inconsistent with the “natural order of things” can disrupt epistemic and existential needs, thus promoting adherence to conspiratorial accounts of the event.

### 1.2. COVID-19 as a Violation of the “Natural Order of Things”

Despite COVID-19 undoubtedly being an upsetting event for most individuals and countries, we advance that there could be some variability in the extent to which the pandemic was perceived as belonging to the “natural order of things”.

In psychological terms, we define the “natural order of things” as a set of expectations based on criteria perceived as universally valid and non-arbitrary, which distinguishes what should and should not happen (see also Pellegrini et al., 2021) [7]. Events that are consistent with such expectations, even when unpleasant and unsought, can be perceived as rightful. On the other hand, events that are inconsistent with the natural order of things are more difficult to be accepted because they are perceived as illegitimate. At the same time, such events signal that the world might not function as it should, and therefore chaos and more unfair outcomes could come.

From a logical point of view, expectations of the natural order of things should be distinguished by predictions of the future. For example, individuals can easily anticipate that, under some circumstances, children may die earlier than their parents, but they may still hold the expectations that this should not happen.

Nevertheless, individuals tend to overlay “is” and “ought” or “facts” and “values” [20,21]. Thereby, a typical evaluation could be as follows: “if something happens, it must have been for good reasons”. It might also follow that people base their predictions on what should be (i.e., the natural order of things). Returning to the earlier example, a person could underestimate the probability of children dying before their parents just because it should not happen according to typical expectations. Thus, events that fall outside of such expectations pose a threat to the perceived ability to make accurate predictions and thus to one’s epistemic ability.

Overall, violations of prescriptive expectations about how the world should function might be perceived as particularly unpleasant because individuals have to face unfair events entailing an unfair and hard-to-predict world.

Mixed positions about the pandemic and its coherence with the natural order of things can be held by different individuals. For many, a pandemic does not particularly violate expectations of the natural order of things because it has afflicted humanity many times in the past. For many others, the sudden disruption and the damage from the pandemic associated with the emergence of a new virus are, per se, a violation of how the world should function.

We advance that, in this second case, individuals might try to reduce the negative psychological consequences of such inconsistency by referring to conspiracy beliefs. Such beliefs may provide simplified explanations of critical situations, thus increasing a sense of control and predictability. In addition, typical conspiracy theories rhetorically attribute the responsibility for the fact that the world is not working according to our expectations to a powerful and evil group of individuals [22]. This involves the world not just being a chaotic place where no basic rules of functioning can be devised. Rather, expectations are well founded but, in special cases, very powerful and evil individuals take actions in their interests, thus disrupting the correspondence between what happens and what should happen. In other words, by attributing responsibility to a special group of malevolent individuals, it is possible to maintain the idea that the natural order of things is not completely and systematically upset.

### 1.3. Overview and Hypotheses

In the present research, we investigated participants’ perception of the pandemic as an event that belongs to the “natural order of things”. This perception should be negatively associated with conspiracy beliefs. In turn, conspiracy beliefs should be associated with a variety of negative and potentially harmful consequences such as the lack of willingness to support the measures taken to deal with the pandemic emergency, a weaker feeling of guilt related to the violation of the anti-COVID-19 government rules, and a lower willingness to undergo vaccination against COVID-19. Conspiracy beliefs are thus expected to play a mediating role between the perception of the natural order of things and the negative consequences of believing in conspiracies. In other words, we expect that considering the COVID-19 pandemic as consistent with the “natural order of things” may reduce the endorsement of conspiracy beliefs and indirectly attenuate the occurrence of potential damaging outcomes.

## 2. Materials and Methods

### 2.1. Participants and Procedure

Data were collected through the recruiting platform of Prolific Academic, during the pandemic emergency in Italy when the first vaccines began to be available (February 2021). Participants received monetary compensation for completing an online questionnaire and thus being enrolled in the study. Specifically, participants were first presented with a short introduction describing the general aims of the research, its correlational nature, and that completing the questionnaire required about 15 min. Then, they were asked to provide their consent to participate. Once participants agreed to participate, they were progressively presented with the measures involved in the research (see the Appendix A) and were asked to carefully read and respond to each of them. Finally, they were thanked for their participation.

The sample size was established using a power analysis specifically designed for mediation models involving a single mediator variable. The analysis was performed using an R application entailing a Monte Carlo simulation approach [23]. We set the conventional power threshold of 0.80 and conservative effect sizes (i.e., expected correlation of 0.15) among the predictor, mediator, and criterion. We also opted for a large number of power analysis replications (5000) and wide coefficient draws per replication (20,000). Based on the settled parameters, a minimum sample size of around 470 participants was needed. Thus, the sample consisted of 565 Italian adults (241 females, Mage = 26.47, SDage = 7.65), all residents in Italy and coming from different geographical areas of the country. The sample’s demographics are summarized in Table 1.

### 2.2. Measures

**Natural Order of Things.** Participants were asked to think about the emergency due to the spread of coronavirus and to indicate their agreement with three items: “It is in the natural order of things that, sooner or later, an epidemic like this occurs”; “Even a terrible event like a pandemic is part of the way in which the world and life work”; “Considering that the pandemic is an event that falls into the overarching scheme of things, it is obvious to think that it is not the first nor the last”. Answers to these items were provided on a 7-point Likert scale ranging from 1 (completely disagree) to 7 (completely agree) and averaged to obtain an overall score (*M* = 5.43, *SD* = 1.12, *α* = 0.84).

**Conspiracy Beliefs.** We implemented the 14-item scale of conspiracy beliefs proposed by Leone et al. [24,25], which asked participants to provide their agreement with a series of conspiratorial explanations linked to an array of issues such as terrorism (e.g., “The so-called Islamic State does not really exist; it is a smoke-screen concocted by Western Governmental Agencies”). To address COVID-19 conspiracy beliefs related to the spread of the pandemic and to the COVID-19 vaccine, we added 7 items to the original 14-item version. Three items investigated conspiratorial explanations of the rise of the COVID-19 pandemic (i.e., “Coronavirus is a U.S. bacteriological weapon to hit China”; “Coronavirus is a Chinese bacteriological weapon accidentally escaped from a laboratory”; “COVID-19 is little more than a flu sold as a global pandemic to terrorize us and rule the world through fear”) and have been taken from Pellegrini et al. [7]. The remaining four items (i.e., “The COVID-19 vaccine allows governments to track and control people”; “The COVID-19 vaccine is harmful, and this is kept hidden”; “Pharmaceutical companies, scientists and academics work together to cover up the dangers of the COVID-19 vaccine”; “The misrepresentation of the efficacy of the COVID-19 vaccine is motivated by profit”) resumed the main conspiratorial thinking about the COVID-19 vaccine. Items were rated on a 5-point Likert scale ranging from 1 (completely disagree) to 5 (completely agree). Responses were averaged (*M* = 1.61, *SD* = 0.59, *α* = 0.94).

**Economic Utilitarian Attitude.** Participants were asked to express their agreement, on a 7-point Likert scale (1 = completely disagree; 7 = completely agree), with three items: “It is necessary to intervene with restrictive measures that protect everyone’s health, even if it will have serious repercussions on the economy of our country”; “It is necessary to keep our country’s economy afloat, even at the cost of sacrificing human lives”; “It is necessary to let the coronavirus run its course, so as to naturally immunize the population (herd immunity)”. After reversing the scores of the first item, we averaged them with those of the other two obtaining an overall score of economic utilitarian attitude (*M* = 2.82, *SD* = 1.15, *α* = 0.71).

**Support for Government Measures.** We measured support for the government’s restrictive measures to tackle the pandemic through four items (e.g., “To what extent are you in favor or against the restrictive measures adopted by the Italian Government to deal with the coronavirus emergency?”). The items were rated on a 7-point Likert scale ranging from 1 (completely against) to 7 (completely in favor) and averaged (*M* = 5.20, *SD* = 1.14, *α* = 0.91).

**Feelings of Guilt.** Feelings of guilt related to the possibility of breaking restrictive rules and potentially causing harm to other people were assessed through four items (e.g., “If I would transgress the restrictive measures adopted by the Italian government to deal with the emergency of the coronavirus, I reproach myself even if nobody suffers from it”). Items were rated on a 7-point Likert scale (1 = completely disagree; 7 = completely agree). To obtain an overall guilt feeling score, items were averaged (*M* = 5.47, *SD* = 1.10, *α* = 0.80).

**Attitudes Towards the COVID-19 Vaccine.** Participants answered six items (e.g., “In general, the COVID-19 vaccine is a good thing”; “I feel uncertain about the safety of the COVID-19 vaccine”) investigating their attitude towards COVID-19 vaccination. Answers were provided on a 7-point Likert scale ranging from 1 (completely disagree) to 7 (completely agree). Items expressing a positive attitude were reversed and then averaged with other items to obtain an overall score indicating negative attitudes towards the COVID-19 vaccine (*M* = 2.58, *SD* = 1.23, *α* = 0.88).

**COVID-19 Vaccine Intention.** Participants’ intention to undergo COVID-19 vaccination was assessed through two items (i.e., “How likely is it that you will undergo the COVID-19 vaccine in the next few months?”; To what extent are you willing to get vaccinated against COVID-19?”). Responses to both items were provided on a 7-point Likert scale ranging from 1 (extremely unlikely; not at all willing) to 7 (extremely likely; totally willing, respectively). Responses from the two items were averaged (*M* = 5.68, *SD* = 1.50, *α* = 0.66).

## 3. Results

**Figure 1:**
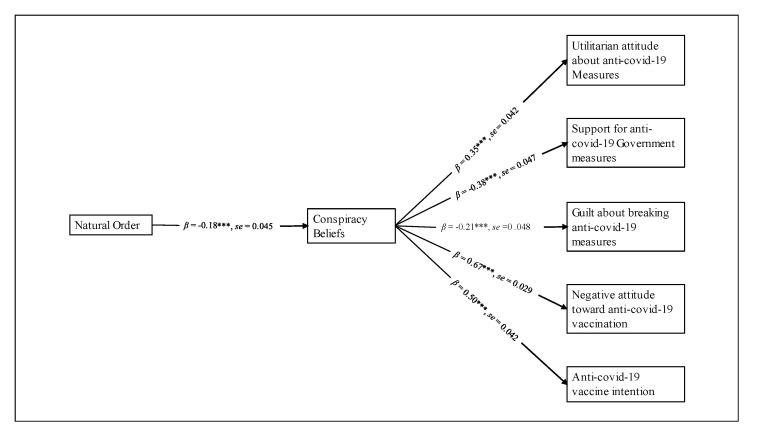
Path analysis model of the investigated variables. *Note*. *** *p* < 0.001.

As a preliminary data overview, we explored intercorrelations among investigated variables (see Table 2). Correlation analyses provided initial support to our expectations, suggesting that emerged associations could be profitably investigated using a parsimonious path analysis model (see Figure 1).

In more detail, we tested a full mediation model where beliefs in a natural order of things could negatively affect the adherence to conspiracy beliefs and, in turn, be indirectly and positively related to support for the anti-COVID-19 government measures, guilt for disregarding these rules and causing harm to other people, and the intention to be vaccinated against COVID-19. Moreover, the mediational pattern was expected to weaken the assumption of an economic utilitarian stance about the adequate political measures for arresting the spread of coronavirus, as well as a negative attitude towards the COVID-19 vaccine.

The proposed total mediation model was performed by a robust maximum likelihood method, with the ‘Huber–White’ correction, and was shown to excellently adhere to the empirical data (*χ*^2^ [5] = 7.66, *p* = 0.18; CFI = 0.997; TLI = 0.989; SRMR = 0.020; RMSEA = 0.031, 90% *CI* = 0.000, 069). We used the ‘Huber–White’ correction since we were interested in testing indirect effects, which are conventionally not normally distributed. The analysis was conducted with *lavaan* [26], an *R* package for structural equation modeling, by using the *RStudio* graphical interface (2022).

As summarized in Table 3, we found a significant and negative association of beliefs in a natural order of things and conspiracy beliefs. This association showed that when participants considered the pandemic emergency as part of an overarching natural order, the need to find a conspiratorial explanation was reduced.

In turn, the proposed mediator (i.e., conspiracy beliefs) was found to be negatively associated with support for the anti-COVID-19 measures, feelings of guilt linked to the eventuality of harming other people and not respecting the containment rules, and anti-COVID-19 vaccination intention (see Figure 1). As expected, conspiracy beliefs were also positively related to the adoption of an economic utilitarian attitude about the political action that should have been taken to hinder the diffusion of coronavirus, as well as to higher negative attitudes towards the COVID-19 vaccine (see Figure 1).

Deepening the analysis of the path model, we detected empirical support in favor of our focal predictions about total mediation effects. Beliefs in a natural order were indirectly and positively related to support for the restrictive measures adopted by the government, to the related feeling of guilt, and to an increased intention to be vaccinated against COVID-19 (see Table 3). On the other hand, beliefs in a natural order were indirectly and negatively associated with an “economy-first” position about the better political strategy to face the pandemic and with negative attitudes towards the COVID-19 vaccine (see Table 3).

## 4. Discussion

COVID-19 poses several different threats to humanity, both in the short and long term. Among these, we focused on the efforts required to protect and restore expectations about reality and the world. When these expectations are invalidated by an overwhelming event such as the pandemic, basic needs can be unmet and conspiracy beliefs can contrast the perceived loss of control and predictability.

Consistent with mounting evidence concerning the negative consequences of endorsing conspiracy beliefs, we found that these beliefs were associated with a wide array of important consequences such as reduced support for anti-COVID-19 measures, reduced guilt in causing harm to other people by not respecting the restrictive measures, and the adoption of utilitarian attitudes that prioritize economic over safety concerns. Perhaps more importantly, endorsement of conspiracy theories was negatively associated with attitudes towards the vaccine and willingness to be vaccinated.

Another key prediction of the present work was also supported. Conspiracy beliefs were negatively associated with the perception that the pandemic falls within the natural order of things. This means that, concerning the pandemic, some individuals did not experience a wide gap between what happened and what should have happened. As a consequence, the world could be perceived as reasonably predictable and manageable and conspiracy beliefs were less central to psychological functioning.

Combining all these findings, a mediation pattern emerged. Perception of the pandemic as belonging (vs. not belonging) to the natural order of things—by reducing the endorsement of beliefs in conspiracies—was positively associated with support for the anti-COVID-19 restrictions, feelings of guilt about breaking these restrictions, and increased intentions to undergo COVID-19 vaccination. Moreover, the mediation pattern between the natural order of things and conspiracy beliefs enfeebled the assumption of a stance prioritizing the economy over people’s health, as well as a negative attitude toward the vaccine against COVID-19. This pattern of results may witness the potential gatekeeper role of existential and epistemic needs of considering an upsetting event as consistent with an overarching scheme of the world functioning, also highlighting the likely practical benefits that this may entail.

The set of questions about conspiracy theories was not limited to COVID-19 but included other domains such as terrorism and other social issues. Thus, the measure refers to a general conspiratorial mindset more than to a specific set of conspiracy beliefs related to COVID-19. It is interesting to note how a specific violation of the natural order of things related to the pandemic was associated with a much broader conspiratorial mindset. This lack of specificity might suggest that conspiracy thinking could be functional to address any inconsistency with the set of expectations about how the world functions. We consider that the attribution of responsibility to an evil and powerful group—a shared principle of many conspiracy beliefs—might be the “active ingredient” for addressing the psychological consequences of inconsistencies with the perceived natural order of things. This speculation could be addressed more precisely in future research.

Present findings add to previous work from Pellegrini et al. [7], who found that the perception of pandemics as belonging to the natural order of things moderated the association between risk perception related to COVID-19 and conspiracy thinking. Here, we showed that perception of coherency with the natural order of things is directly associated with conspiracy thinking. In other words, a person does not need to feel a direct threat from COVID-19 (e.g., contagion, disease, death) as a prerequisite to refer to conspirations. Perceiving that an event is inconsistent with the natural order of things might interfere directly with existential and epistemic needs in a way that is sufficient to trigger conspiracy thinking, as a compensatory mechanism. Future research could examine more precisely this hypothesized path since in the present work we did not measure directly epistemic and existential needs.

The present research also provides hints concerning how to reduce and prevent individuals’ appeal to conspiracy thinking. More specifically, our findings suggest that a key variable is how the pandemic is framed within a larger worldview (i.e., natural order of things). In the present case, we considered individual differences in the belief that the pandemic belongs to the natural order of things. However, building on present findings, it could be worth to examine whether social interventions designed to promote this belief might efficaciously reduce the endorsement of conspiracy and, hopefully, their negative consequences.

The main weakness of the present study is the cross-sectional nature of the data, which limit us from making causal inferences. As indicated by Maxwell et al. [27], cross-sectional data are not entirely recommended for performing mediation. However, the main aim of this work was to establish a model of associations that operate through indirect paths (i.e., conspiracy beliefs) and this can be achieved satisfactorily even with correlational data. In the future, experimental research would be needed to test whether interventions to reduce conspiracy thinking based on present findings can be effective.

Another limitation may be found in the narrowed generalizability of the results. The research was carried out by recruiting a community sample, which is not certainly representative of the Italian population. Nevertheless, the large sample size provided the analyses with sufficient statistical power, allowing us to be reasonably confident of the reliability and soundness of the emerged associations. Future studies based on representative samples could provide more stable evidence about the generalizability of our results.

Furthermore, since the present study is based on data collected in Italy, future studies may endeavor to provide further support regarding the generalizability of our conclusions focusing on different national contexts.

## 5. Conclusions

Reporting of pandemics in the past centuries suggest that these events have been perceived, throughout history, as a violation of the natural order of things. In many cases, people tried to protect and restore their expectations about the order of things by attributing the responsibility of the events to wrongful actions or intentions coming from their own in-group (i.e., pandemics as a divine punishment for their behavior) or other evil groups. Conspiracy thinking is an instance of this latter option, and it is motivated by basic needs. It is however important to reduce the appeal of these compensatory strategies because their costs greatly mismatch their benefits. Our results highlight that construing the pandemic as coherent with the natural order of things is associated with reduced need for conspiratorial explanations and the related set of beneficial effects on an array of outcomes pertaining to the promotion of individual and collective well-being. From this perspective, our results could represent a cue from which to draw indications for the implementation of interventions aimed at the psychosocial management of shocking events, preventing their potential negative consequences, and encouraging responsible behaviors to deal with them.

## Figures and Tables

**Table 1:** Sample’s Demographics.

Variables	*Frequency*	*Percentage*
**Gender**		
Male	324	57.3
Female	241	42.7
**Nationality**		
Italian	563	99.6
Other European nationality	1	0.2
Other non-European nationality	1	0.2
**Country of residence**		
Italy	565	100
**Geographical area of residence**		
North	132	43.4
Center	132	23.4
South	122	21.6
Islands	66	11.7
**Education**		
Lower secondary school diploma	14	2.5
High school diploma	317	56.1
Degree	207	36.6
PhD/Postgraduate specialization	27	4.8
**Employment condition**		
Employed	116	20.6
Self-employed	40	7.6
Unemployed or homemaker	80	14.2
Other occupations	18	3.2
Student	311	55

**Table 2:** Correlations.

Variables	1	2	3	4	5	6
1. Natural Order	-					
2. Conspiracy Beliefs	−0.18 ***	-				
3. Utilitarian Attitude	0.01	0.35 ***	-			
4. Support government measure	0.06	−0.38 ***	−0.56 ***	-		
5. Guilt Feelings	−0.02	−0.21 ***	−0.40 ***	0.45 ***	-	
6. Vaccine Negative Attitude	−0.16 ***	0.67 ***	0.37 ***	−0.42 ***	−0.24 ***	-
7. Vaccine Intention	0.11 **	−0.50 ***	−0.33 ***	0.40 ***	0.30 ***	−0.68 ***

*Note*. ** *p* < 0.01, *** *p* < 0.001.

**Table 3:** Results of Path Analysis Model.

Mediator Model						95% *CI*	
Predictor	Mediator	*β*	*se*	*z*	*p*	*Lower*	*Upper*
Natural Order	Conspiracy Beliefs	−0.18	0.045	−4.02	<0.001	−0.268	−0.092
Outcome Model						95% *CI*	
Mediator	Criteria	*β*	*se*	*z*	*p*	*Lower*	*Upper*
Conspiracy Beliefs	Utilitarian Attitude	0.35	0.042	8.26	<0.001	0.267	0.433
	Support government measure	−0.38	0.047	−7.92	<0.001	−0.469	−0.283
	Guilt Feelings	−0.21	0.048	−4.31	<0.001	−0.301	−0.113
	Vaccine Negative Attitude	0.67	0.029	23.13	<0.001	0.610	0.723
	Vaccine Intention	−0.50	0.042	−11.99	<0.001	−0.587	−0.422
Indirect Effects						*95% CI*	
	Criteria	*β*	*se*	*z*	*p*	*Lower*	*Upper*
	Utilitarian Attitude	−0.06	0.017	−3.71	<0.001	−0.096	−0.030
	Support government measure	0.07	0.019	3.48	0.001	0.029	0.106
	Guilt Feelings	0.04	0.013	2.90	0.004	0.012	0.062
	Vaccine Negative Attitude	−0.12	0.031	−3.90	<0.001	−0.180	−0.060
	Vaccine Intention	0.09	0.024	3.74	<0.001	0.043	0.138

## Data Availability

The data that support the findings of this study are available from the corresponding author upon reasonable request.

## Appendix A

**Questionnaire**

**Introduction and informed consent**

Gentile Partecipante,

Questa ricerca si propone di studiare le opinioni delle persone su alcune questioni quotidiane e su alcune vicende politiche. Grazie alla sua collaborazione potremo raccogliere informazioni importanti per comprendere qualcosa in più sulle opinioni delle persone su alcuni importanti problemi.

Le chiediamo di rispondere nel modo più sincero possibile alle domande del questionario. Le chiediamo inoltre di rispondere a tutte le domande.

Il questionario è totalmente anonimo. Ai sensi delle norme vigenti, i dati da lei forniti, trasformati in numeri da ricercatori vincolati dal segreto professionale, saranno trattati solo in forma statistica per scopi scientifici.

Consenso informato: sono consapevole che il consenso è espressione di una libera decisione, non influenzata da promesse di benefici economici o di altra natura, né da obblighi nei confronti del Ricercatore responsabile dello studio. Sono consapevole di essere libero/a di ritirarmi dallo studio in qualsiasi momento io lo desideri. Sono consapevole, inoltre, di non avere l’obbligo di motivare la mia decisione di ritirarmi dallo studio. Mi è stata data l’opportunità di leggere le informazioni contenute nella parte informativa di questo documento. Ho compreso tutte le informazioni e ho avuto il tempo sufficiente per prendere in considerazione la mia partecipazione a questo studio.

In conseguenza di ciò:

○ Acconsento a partecipare allo studio
○ Non acconsento a partecipare allo studio

DEMOGRAPHICS

La preghiamo di indicare la Nazione in cui attualmente risiede:

○ Italia
○ Altra Nazione Europea
○ Altra Nazione Extraeuropea

La preghiamo di indicare la sua Nazionalità:

○ Italiana
○ Altra Nazionalità Europea
○ Altra Nazionalità Extraeuropea

La preghiamo di indicare la zona geografica in cui attualmente risiede

○ Nord
○ Centro
○ Sud
○ Isole

Genere:

○ Maschio
○ Femmina

Età:

scrivere la propria età in cifre (ad esempio, 30).

________________________________________________________________

Titolo di studio:

○ Nessun titolo di studio
○ Licenza di scuola elementare
○ Licenza di scuola media
○ Diploma di scuola superiore
○ Laurea
○ Dottorato/Specializzazione post-laurea

Condizione lavorativa attuale:

○ Studente
○ Operaio/a
○ Impiegato/a
○ Commerciante
○ Imprenditore
○ Libero professionista
○ Casalinga/o
○ Pensionato/a
○ Disoccupato/a
○ Altro

**NATURAL ORDER OF THINGS**

**1 = Totally disagree 7 = Totally agree**

Pensando alla situazione di emergenza dovuta alla diffusione del coronavirus in Italia, indichi il suo grado di accordo con le seguenti affermazioni:	
1. È nell’ordine naturale delle cose che, prima o poi, un’epidemia come questa si verifichi.	1 2 3 4 5 6 7
2. Anche un evento terribile come una pandemia è parte del modo in cui funziona il mondo e la vita.	1 2 3 4 5 6 7
3. Considerando che l’epidemia è un evento che ricade nel grande schema delle cose, è ovvio pensare che non sia la prima e neanche l’ultima.	1 2 3 4 5 6 7

**CONSPIRACY BELIEFS**

**1 = Completely disagree 5 = Completely agree**

1. Il governo degli stati uniti ha permesso che gli attacchi di Settembre del 2001 avessero luogo per poter poi scatenare le guerre in Afghanistan e in Iraq.	1 2 3 4 5
2. Esiste già una cura definitiva per il cancro, ma le industrie del farmaco preferiscono tenerla segreta e continuare a guadagnare soldi vendendo le chemioterapie.	1 2 3 4 5
3. La crisi finanziaria del 2008 e l’attuale crisi economica sono il risultato di azioni deliberate da parte di banchieri, finanzieri e multinazionali.	1 2 3 4 5
4. Le crisi finanziarie degli ultimi anni sono state provocate deliberatamente dai poteri politici e finanziari.	1 2 3 4 5
5. Il governo degli Stati Uniti sapeva degli attacchi organizzati l’11 Settembre e ha deciso di non fare nulla per prevenirli.	1 2 3 4 5
6. Le multinazionali che producono bibite gassate le arricchiscono con sostanze che causano dipendenza.	1 2 3 4 5
7. Molte malattie infettive, come l’Ebola, sono deliberatamente diffuse da governi e multinazionali del farmaco per scopi economici.	1 2 3 4 5
8. Il cosiddetto ISIS (Stato Islamico) non esiste, ma è solo una creazione dei servizi segreti occidentali.	1 2 3 4 5
9. La lobby gay ha un piano per aumentare il numero di omosessuali diffondendo la “teoria del gender”.	1 2 3 4 5
10. L’assassinio del giudice Giacomini è stato portato a termine dalla mafia con la complicità dello Stato italiano.	1 2 3 4 5
11. I vaccini sono inutili e dannosi, vengono somministrati solo per favorire gli interessi delle case farmaceutiche.	1 2 3 4 5
12. Tramite le scie chimiche gruppi di potere intendono avvelenare l’aria o manipolare il clima.	1 2 3 4 5
13. Alcuni gruppi di pochi potenti (Bilderberg, Trilaterale, Banca Rotschild, Massoneria) manipolano i destini del mondo.	1 2 3 4 5
14. I rischi dei vaccini sono di molto superiori ai loro ipotetici benefici.	1 2 3 4 5
15. Esiste un piano delle élite politiche per mischiare i migranti con gli europei.	1 2 3 4 5
16. I fenomeni migratori sono parte di un’operazione di sostituzione etnica coordinata dall’Europa.	1 2 3 4 5
17. Il coronavirus è un’arma batteriologica statunitense per colpire la Cina.	1 2 3 4 5
18. Il coronavirus è un’arma batteriologica cinese sfuggita per errore da un laboratorio.	1 2 3 4 5
19. Il coronavirus è poco più di un’influenza spacciata per epidemia globale per terrorizzarci e governare il mondo tramite la paura.	1 2 3 4 5
20. Il vaccino anti COVID-19 consente ai governi di tracciare e controllare le persone.	1 2 3 4 5
21. Il vaccino anti COVID-19 è dannoso e ciò viene tenuto nascosto.	1 2 3 4 5
22. Aziende farmaceutiche, scienziati e accademici lavorano insieme per coprire i pericoli del vaccino anti COVID-19.	1 2 3 4 5
23. La falsa rappresentazione dell’efficacia del vaccino anti COVID-19 è motivata dal profitto.	1 2 3 4 5

**ECONOMIC UTILITARIAN ATTITUDE**

**1 = Completely disagree 7 = Completely agree**

Gli economisti italiani hanno stimato un calo del Prodotto Interno Lordo (PIL) del nostro Paese compreso tra i 3 e gli 8 punti percentuali a causa dell’emergenza coronavirus. Sulla base di questa previsione, la preghiamo di indicare il suo grado di accordo con le seguenti misure politiche per affrontare l’emergenza coronavirus:	
1. È necessario intervenire con misure restrittive che tutelino la salute di tutti, anche se avrà delle ripercussioni gravi sull’economia del nostro paese.	1 2 3 4 5 6 7
2. È necessario tenere a galla l’economia del nostro Paese, anche al costo di sacrificare delle vite umane.	1 2 3 4 5 6 7
3. È necessario lasciare che il coronavirus faccia il suo corso, così da immunizzare naturalmente la popolazione (immunità di gregge).	1 2 3 4 5 6 7

**GUILT FEELINGS**

**1 = Completely disagree 7 = Completely agree**

La preghiamo di indicare il suo grado di accordo con le seguenti affermazioni:	
1. Se mi capita di trasgredire le misure restrittive adottate dal governo italiano per fronteggiare l’emergenza del coronavirus, mi rimprovero anche se nessuno ne soffre.	1 2 3 4 5 6 7
2. Se non agisco in conformità con le misure restrittive adottate dal governo italiano per fronteggiare l’emergenza del coronavirus, mi sento in colpa.	1 2 3 4 5 6 7
3. Pensando all’emergenza coronavirus, se dovessi provocare un danno a qualcuno mi sentirei in colpa per il male arrecato.	1 2 3 4 5 6 7
4. Pensando al coronavirus, quando vedo qualcuno che soffre provo pena per lui.	1 2 3 4 5 6 7

**SUPPORT FOR GOVERNMEANT MEASURES**

In che misura è favorevole o contrario alle misure restrittive adottate dal governo italiano per fronteggiare l’emergenza coronavirus?

	**Del Tutto Contrario**	**Contrario**	**Abbastanza Contrario**	**Né Contrario Né Favorevole**	**Abbastanza Favorevole**	**Favorevole**	**Del Tutto Favorevole**
5							
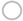

In che misura ritiene illegittime o legittime le misure restrittive adottate dal governo italiano per fronteggiare l’emergenza coronavirus?

	**Del Tutto Illegittime**	**Illegittime**	**Abbastanza Illegittime**	**Né Illegittime Né Legittime**	**Abbastanza Legittime**	**Legittime**	**Del Tutto Legittime**
5							

In che misura ritiene ingiuste o giuste le misure restrittive adottate dal governo italiano per fronteggiare l’emergenza coronavirus?

	**Del Tutto Ingiuste**	**Ingiuste**	**Abbastanza Ingiuste**	**Né Ingiuste Né Giuste**	**Abbastanza Giuste**	**Giuste**	**Del Tutto Giuste**
5							

In che misura ritiene ingiustificabili o giustificabili le misure restrittive adottate dal governo italiano per fronteggiare l’emergenza coronavirus?

	**Del Tutto Ingiustificabili**	**Ingiustificabili**	**Abbastanza Ingiustificabili**	**Né Ingiustificabili Né Giustificabili**	**Abbastanza Giustificabili**	**Giustificabili**	**Del Tutto Giustificabili**
5							

**ATTITUDE TOWARD ANTI-COVID-19 VACCINE**

**1 = Completely disagree 7 = Completely agree**

Le seguenti affermazioni riguardano la sua opinione circa il vaccino anti COVID-19. La preghiamo di indicare il suo grado di accordo con ciascuna di esse:	
1. Sono preoccupato dei potenziali effetti collaterali del vaccino anti COVID-19.	1 2 3 4 5 6 7
2. Mi sento incerto sulla sicurezza del vaccino anti COVID-19.	1 2 3 4 5 6 7
3. Il vaccino anti COVID-19 è stato sviluppato troppo velocemente per essere sicuri di vaccinarsi.	1 2 3 4 5 6 7
4. In generale, il vaccino anti COVID-19 è una buona cosa.	1 2 3 4 5 6 7
5. Il vaccino anti COVID-19 ci permetterà di tornare alla “normalità”.	1 2 3 4 5 6 7
6. Dubito che il vaccino anti COVID-19 sia il modo giusto per raggiungere l’immunità di gregge.	1 2 3 4 5 6 7

**ANTI-COVID-19 VACCINE INTENTION**

Quanto è probabile che nei prossimi mesi usufruirai del vaccino anti COVID-19?

Estremamente improbabile	Moderatamente improbabile	Leggermente improbabile	Né probabile né improbabile	Leggermente probabile	Moderatamente probabile	Estremamente probabile
						

In che misura sei disposto a vaccinarti contro il COVID-19?

Per niente disposto						Del tutto disposto
						
